# Diversity and Antibacterial Activities of Fungi Derived from the Gorgonian *Echinogorgia rebekka* from the South China Sea

**DOI:** 10.3390/md9081379

**Published:** 2011-08-12

**Authors:** Ya-Nan Wang, Chang-Lun Shao, Cai-Juan Zheng, Yi-Yan Chen, Chang-Yun Wang

**Affiliations:** 1 Key Laboratory of Marine Drugs, Ministry of Education, School of Medicine and Pharmacy, Ocean University of China, Qingdao 266003, China; E-Mails: yanan_wang@sina.com (Y.-N.W.); shaochanglun@ouc.edu.cn (C.-L.S.); caijuan2002@163.com (C.-J.Z.); yiyanchen80@hotmail.com (Y.-Y.C.); 2 Department of Biology, Qingdao University, Qingdao 266071, China

**Keywords:** gorgonian coral, *Echinogorgia rebekka*, fungi, diversity, antibacterial activity

## Abstract

The diversity of symbiotic fungi associated with the gorgonian coral *Echinogorgia rebekka* from the Weizhou coral reef in the South China Sea was investigated. Combined with morphologic traits, ITS-rDNA sequences revealed 18 fungal strains from this gorgonian. All of the 18 fungi belonged to the phylum Ascomycota and were distributed among seven genera in five orders: Eurotiales (*Aspergillus* and *Penicillium*), Pleosporales (*Alternaria*), Capnodiales (*Cladosporium*), Trichosphaeriales (*Nigrospora*) and Hypocreales (*Hypocrea* and *Nectria*). Antibacterial activities of these fungal strains were investigated with five pathogenic bacteria. All of the 18 fungal strains displayed different levels of antibacterial activities, most of which exhibited moderate to high antibacterial activities to the Gram-positive pathogens *Staphylococcus aureus* and *Micrococcus tetragenus*, and showed relatively low bioactivities to other three pathogenic bacteria. Several fungal strains in the genera *Penicillium* and *Cladosporium* with strong antibacterial activities provide potential for further research on isolation of bioactive secondary metabolites.

## 1. Introduction

Coral reefs are widely recognized as one of the most diverse ecosystems on Earth, but little attention has been paid to the microbial communities associated with coral reefs. Complex microbial communities are known to be extremely important members of many ecosystems, and also have significant influence over coral reef ecosystems [1–3]. In fact, the coral reef provides a structurally and environmentally complex array of habitats supporting a broad microbial diversity that influences both host physiology and ultimately ecosystem processes [4]. Marine microbes have been shown to produce prolific novel bioactive natural products that are not found in terrestrial strains [5–7], and symbiotic microbes are believed to be the actual producers or take part in the biosynthesis of some bioactive marine secondary metabolites isolated from the marine organism hosts [8,9]. The large number of symbiotic microbes in corals is considered to be a promising source of novel drugs which have been largely unexplored [10,11]. Limited information is known about the microbial diversity associated with marine coral reefs, despite the vital role that microorganisms may play in coral reef ecosystems. As part of our ongoing investigation on antibacterial symbiotic microorganisms from marine invertebrates in the South China Sea, the fungi isolated from a gorgonian *Echinogorgia rebekka* attracted our attention because the fungal strains exhibited relatively high diversity and the ethyl acetate (EtOAc) extracts of the fungal cultures showed rather high antibacterial activities. Herein we report the fungal isolation and identification as well as the antibacterial activities of fungal extracts from this gorgonian. The diversity of symbiotic fungi was examined based on morphologic observations and phylogenetic relationships of ITS-rDNA sequences of the 18 isolated fungal strains. Antibacterial activities of the fungal extracts were investigated with five pathogenic bacteria including *Escherichia coli* ATCC25922, *S. aureus* ATCC6538P, *Bacillus subtilis* 20019, *M. tetragenus* ATCC13623 and *Vibrio anguillarum* ATCC19019.

## 2. Results and Discussions

### 2.1. Diversity of Culturable Fungi Derived from the Gorgonian *Echinogorgia rebekka*

**Figure 1.**
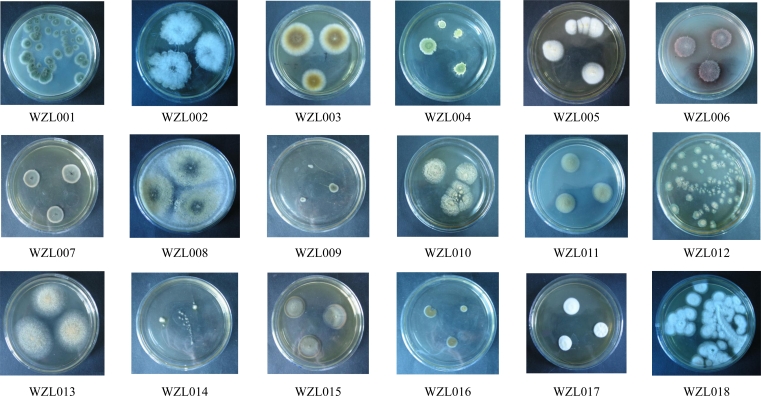
Morphological photos of 18 gorgonian-derived fungal strains (WZL001–WZL018).

Cultivation of fungi from the tissue of the gorgonian coral *Echinogorgia rebekka* yielded a total of 53 isolates. Redundant isolates were excluded under the guidance of observation on morphological characteristics and 18 distinct isolates were identified (WZL001–WZL018; Figure 1, Table 1). There was no dominant morphotypes covering most of the strains, but strains from *Pennicillium* sp., *Aspergillus* sp. and *Cladosporium* sp. account for a large proportion of the 53 isolates. The 18 isolates were cultured for genomic DNA extraction and sequencing analysis. Of the 18 strains, most were distinct from each other and could be identified in morphology and/or in DNA sequences, with the exception of the WZL007 and WZL009 (Figure 1, Table 1). These two strains were highly similar in the sequences of ITS region, differing by only one nucleotide, and they were also similar in clonal and microscopic morphology (Figure 1). We cultivated these two isolates under the same culture conditions for a long period of time, and it was consistent that the WZL007 could produce conidia spores while WZL009 could not breed spores. This distinguishable characteristic in production indicates that these two isolates likely belong to different species.

**Figure 2.**
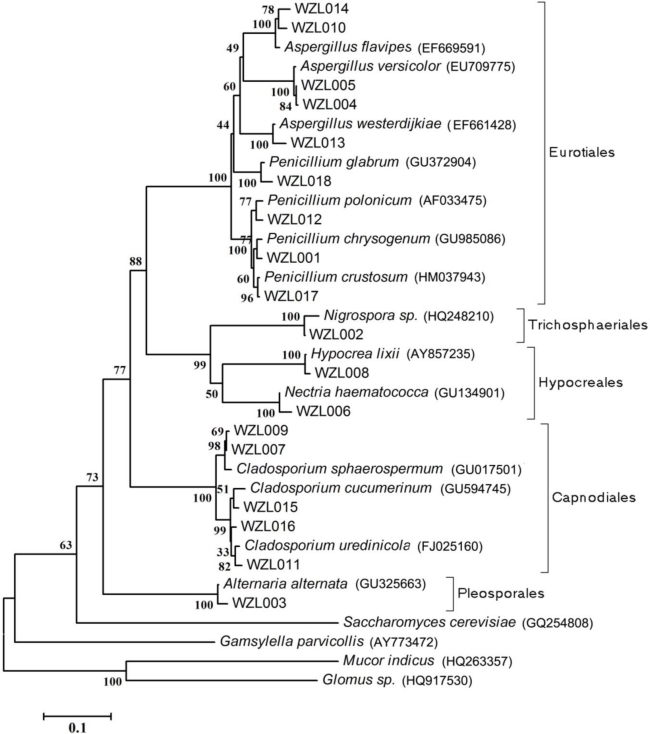
Phylogenetic tree of partial ITS-rDNA sequences of gorgonian-derived fungal strains. Reference sequences were downloaded from NCBI with the accession numbers indicated in parentheses.

The rooted NJ tree of partial ITS-rDNA gene sequences of fungal isolates is shown in Figure 2. In total, seven genera of ascomycetes were recognized from 18 fungal strains including *Aspergillus* (Eurotiales, Eurotiomycetes), *Peniillium* (Eurotiales, Eurotiomycetes), *Alternaria* (Pleosporales, Dothideomycetes), *Cladosporium* (Capnodiales, Dothideomycetes), *Nigrospora* (Trichosphaeriales, Sordariomycetes), *Hypocrea* (Hypocreales, Sordariomycetes) and *Nectria* (Hypocreales, Sordariomycetes) (Figure 2). The most abundant fungi were observed in the genera *Aspergillus* and *Cladosporium* (5 strains for each). Relatively high diversity, *i.e.*, a significant number of different strains found in a genus, was also detected in the genera *Penicillum* and *Cladosporium* (4 strains for each). For the other four genera, only one strain was found (Table 2, Figure 2).

The 18 fungal strains derived from the gorgonian *Echinogorgia rebekka* were shown to be distributed in seven genera of the phylum Ascomycota. In contrast with the the *Nectria* and *Hypocrea*, the other five fungal genera were known to be marine-derived (*Nigrospora* [12]; *Alternaria* [13]; *Aspergillus*, *Penicillium*, and *Cladosporium* [14]). The ascomycetes represent those fungi that are readily cultivable and could be easily recovered when culture-dependent techniques are applied [15]. Representative strains of these fungi have previously been isolated from marine algae, seagrasses, sponges and cnidarians [16]. The ascomycetes have been reported as invertebrate symbiotic fungi and as a prolific source of biologically active secondary metabolites, such as polyketides with antitumoral and/or antimicrobial activities [15–18]. Our investigation revealed that the diversity of symbiotic fungi (*Aspergillus*, 5 strains; *Penicillium*, 4 strains; *Cladosporium*, 5 strains) isolated from the gorgonian *Echinogorgia rebekka* was high.

### 2.2. Antibacterial Activities of the Extracts from Fungal Broth and Mycelia

Antibacterial activities of the extracts from the fermentation supernatant and mycelia of 18 isolated fungi were assayed by using four terrestrial pathogenic bacteria (*Escherichia coli* ATCC25922, *S. aureus* ATCC6538P, *Bacillus subtilis* 20019 and *M. tetragenus* ATCC13623) and one marine pathogenic bacterium (*Vibrio anguillarum* ATCC19019). *E. coli*, *S. aureus*, *B. subtilis*, and *M. tetragenus* are common pathogens and model bacteria for this type of work. *V. anguillarum* is a harmful bacterium in aquaculture. The inhibition activities to these 5 bacterial can provide useful data to find promising natural products candidates. All of the 18 fungi showed different levels of antibacterial activities to at least one pathogen with their fermentation broth and/or mycelia (Table 2). Out of the 18 fungal strains, 12 were found to show moderate to high level of antibacterial activities to *S. aureus*, while 9 had moderate to very high activities to *M. tetragenus* (Table 2). It is worth pointing out that the mycelia EtOAc extract of the *Penicillium* strain WZL018 displayed exceptionally high antibacterial activities to *M. tetragenus* (growth inhibition diameters: larger than 15 mm). In addition, the mycelia EtOAc extract of the *Penicillium* WZL017 and the *Cladosporium* strain WZL015 exhibited high activities to the pathogen *B. subtilis* with growth inhibition diameters between 10 and 15 mm, while the broth EtOAc extract of *Cladosporium* strain WZL009 displayed similar activities to the pathogen *S. aureus* (Table 2). Microbes can secrete a wide variety of metabolites including intracellular and extracellular products [19]. From the data in the present study, we found that some broth and/or mycelia EtOAc extracts of fungi have the antibacterial activities. This suggested that different fungi could produce intracellular bioactive metabolites or secrete extracellular bioactive compounds. In addition, the results indicated that the fungal extracts exhibited higher inhibition activities to Gram-positive bacteria, such as *B. subtilis*, *S. aureus* and *M. tetragenus*, than to Gram-negative bacteria, which might be a result of different cell wall compositions of Gram-positive and Gram-negative bacteria. Many diseases are known to be caused by gram-positive, such as the infection of respiratory tract and skin. The gram-positive bacteria can produce exotoxin which can infect human bodies. The fungi with better bioactivity toward gram-positive bacteria will be a possible source of medicine to treat these diseases. Our results indicate that the gorgonian-derived fungi isolated in this study, especially the *Penicillium* strains WZL018, WZL017, and the *Cladosporium* strains WZL015, WZL009, exhibited potential for the isolation of antibacterial natural products.

## 3. Experimental Section

### 3.1. Coral Materials

The sample of a gorgonian coral *Echinogorgia rebekka* was collected from the Weizhou coral reef in the South China Sea in September 2008. The coral sample was transferred directly to a sterile plastic bag without seawater. Seawater samples from the collection sites were collected to serve as controls. The samples were stored immediately at 4 °C and sent to the laboratory as soon as possible for the isolation of fungi. Latex gloves were worn during collection.

### 3.2. Fungi Isolation

The coral sample was rinsed three times with sterile artificial seawater to get rid of nonspecific fungal propagules that stick to the coral surface from seawater colony, and the surface of the coral was disinfected with 70% ethanol [20]. Then the coral was cut into small pieces and homogenized using a blender containing 20 mL sterile natural seawater under aseptic conditions. The resulting homogenate was diluted with sterile seawater at three dilutions (1:10, 1:100, and 1:1000). For fungi cultivation, 100 μL of each dilution was plated in quadruplicate onto the four plates of the following medium: potato dextrose agar (PDA) medium (200 g of washed but unpeeled potatoes slices were boiled in 1 L seawater for 30 min, 20 g dextrose and 20 g agar powder was then added into the potato soup), Rose Bengal Medium (RBM) (peptone 5 g, glucose 10 g, KH_2_PO_4_ 1 g, MgSO_4_·7H_2_O 0.5 g, Rose Bengal 0.033 g, agar 20 g, seawater 1 L), Czapek-Dox medium (NaNO_3_ 3 g, KCl 0.5 g, K_2_HPO_4_ 0.1 g, MgSO_4_·7H_2_O 0.5 g, FeSO_4_ 0.01 g, sucrose 30 g, agar 20 g, seawater 1 L, pH 6.7), and Luria Bertani (LB) agar medium. The antibiotics of penicillin and streptomycin (100 mg/mL for each) were added to each plate described above. The plates were incubated at 28 °C for 1–3 weeks until the morphology of fungi could be distinguished. The fungi isolates were identified morphologically according to Wei’s morphological criteria [21]. The morphological traits (e.g., morphology and color of spore and mycelia) of the pure fungal colonies were examined by microscopy to exclude redundant fungal isolates and were used to group the strains into different morphotypes. Photos were taken for distinct isolates. Each isolate was picked and transferred onto a new corresponding agar plate containing PDA, RBM, Czapek-Dox, or LB medium with penicillin and streptomycin. The resulting plates were incubated at 28 °C for pure culture.

### 3.3. Extraction of Genome DNA from Cultured Fungi

The distinct fungi isolates described in the above paragraph were cultured in GPY broth (glucose 20 g, yeast extract paste 10 g, peptone 20 g, dissolved in 1 L seawater, pH 6.5) at 28 °C for 2–5 days. The mycelia was harvested by using vacuum filtration and dried with two layers of paper towel. The resulting mycelial mat was ground into powder with liquid nitrogen.

The fungal DNA was extracted according to the procedure described by Fredricks with modifications [22]. In brief, the mycelial powder was transferred to a 1.5 mL Eppendorf tube containing 500 μL TE buffer (10 mM Tris, 0.1 mM EDTA, pH 7.3). Lysozyme was then added, and the mixture was incubated at 37 °C for 30 min. Later, 10% SDS (1:10, w:v) and proteinase K (1:100, w:v) were added and incubated at 56 °C for 3 h. An equal volume of buffer equilibrated phenol was added followed by brief mixing and the mixture was centrifugation at 12,000 rpm for 10 min at 4 °C. The upper aqueous phase was gently transferred to a new tube, and an equal volume of chloroform/phenol (1:1, v:v) was added. The mixture was separated by centrifugation at 12,000 rpm for 10 min and the aqueous phase was removed. Then, 3 mol/L sodium acetate (1:10, v:v) and RNaseA were added to remove RNA. DNA was precipitated by adding 2.5 volumes of absolute ethanol. The DNA pellet was washed with 75% ethanol twice and resuspended in 50 μL of sterile water to be dissolved.

### 3.4. Amplification of Fungal ITS-rDNA Fragments

The genomic DNA was used as the template to amplify fungal ITS-rDNA fragments using the primers ITS1 (5′-TCCGTAGGTGAACCTGCG-3′) and ITS4 (5′-TCCTCCGCTTATTGATATGC-3′) [23] which were synthesized by Shanghai Sangon Biological Technology and Service Co., Ltd. The reaction mixture for PCR amplification contained 5 μL of 10× reaction buffer with 15 mM MgCl_2_ (TaKaRa), 2 μL of 2.5 mM dNTPs, 0.5 μL of 10 μM each primer, 4 μL of fungal DNA, 0.3 μL of Taq DNA polymerase (5 U·μL^−1^, TaKaRa), and 39.7 μL of H_2_O. PCR conditions included an initial denaturation at 94 °C for 5 min followed by 32 cycles of denaturation at 94 °C for 50 s, annealing at 52.5 °C for 50 s, and elongation at 72 °C for 1 min, with a final elongation at 72 °C for 10 min. PCR products were purified using the Agarose Gel DNA Purification Kit (TaKaRa) and sequenced in Shanghai Sangon Biological Engineering Technology and Service Co., Ltd.

### 3.5. Sequence and Phylogenetic Analysis

For preliminary identification, sequences of fungal ITS-rDNA regions obtained from the gorgonian coral *Echinogorgia rebekka* were compared with related sequences in NCBI [24]. Fungal ITS-rDNA sequences acquired in this study were edited and aligned with the best n-BLAST hits from GenBank in the Clustal X (version 1.83) program [25], and further manually adjusted using BioEdit software [26]. The sequences obtained in this study were deposited in the GenBank/EMBL/DDBJ under the accession numbers HQ588304–HQ588321. The program MEGA 5 [27] was applied to calculate the base composition of the fungal sequences. To analyze the relationship between the fungal species, MEGA 5 was also used to reconstruct phylogenetic trees using the neighbor-joining (NJ) method. A maximum likelihood statistical method implemented in MEGA 5 was first used to determine the best-fit nucleotide substitution models for ITS sequences based on Bayesian Information Criterion (BIC); BIC values revealed that the best substitution model was K2 + G model [28]. Then this model was employed to reconstruct phylogenetic trees with 10,000 bootstrap replicates. ITS sequences of related fungi were downloaded from NCBI as references.

### 3.6. Assay of Antibacterial Activity

The fungi was inoculated into 500 mL Erlenmeyer flasks containing 150 mL of liquid medium (glucose 10 g, yeast extract paste 1 g, peptone 3 g, dissolved in 1 L seawater, pH 6.5), followed by shaking incubation at 28 °C with 165 rpm for nine days. The fermented whole broth was filtered through cheese cloth to separate the supernatant and the mycelia. The supernatant layer was concentrated to about a quarter of the original volume under reduced pressure, and then extracted with ethyl acetate (EtOAc) three times. The EtOAc phase was evaporated to gain EtOAc extract. The mycelia were extracted firstly with acetone three times, and the solvent was evaporated *in vacuo* to give a residue, which was extracted with EtOAc three times to yield another EtOAc extract. The obtained EtOAc extracts were stored after freeze drying.

Five pathogenic microorganisms were used for the antibacterial assay, including four terrestrial bacteria (*E. coli* ATCC25922, *S. aureus* ATCC6538P, *B. subtilis* 20019, *M. tetragenus* ATCC13623) and one marine bacterium *Vibrio anguillarum* ATCC19019. After overnight culture, each tested bacterium was adjusted to 2 × 10^8^–5 × 10^8^ colony forming units per mL, and 0.1 mL of such a culture was spread on medium of Petri dishes (Φ 9 cm). *V. anguillarum* were grown in Tryptic Soy Brothor Agar (TSB, TSA) and others were grown in Nutrient Agar (NA). Holes (Φ 6 mm) were then drilled on the agar plates. The lyophilized EtOAc extract described in the above paragraph was dissolved in DMSO with a final concentration of 100 μg/mL and used as a test solution. And 5 μL of the test solution was added to each hole on the plates. The dishes were incubated at 37 °C for 16–24 h. The diameters of the inhibition zone surrounding the holes were measured. DMSO and streptomycin were used as negative control and positive control, respectively. The experiments were carried out in triplicates.

## 4. Conclusions

A total of 18 symbiotic Ascomycota fungi were isolated from the gorgonian coral *Echinogorgia rebekka*. They were distributed among seven genera in five orders. These ascomycetes displayed different levels of antibacterial activities to pathogenic bacteria, and some strains in the genera *Penicillium* and *Cladosporium* showed strong growth inhibition to *B. subtilis*, *S. aureus* and *M. tetragenus.* The combined data gathered from this investigation contribute to broadening the knowledge of microbial diversity and antibacterial bioactivities associated with corals. As corals are an important source of marine bioactive natural products and drugs, and the coral resources are decreasing and limited, the isolation of symbiotic microbes from the gorgonian corals and the screening for bioactivities will have important significance for further research to discover new bioactive natural products. Meanwhile, due to the emergence of antibiotic resistance of traditional natural products and drugs, there is a pressing need for the development of novel antimicrobial therapies. Recently, researchers have invested their interest in the development of antimicrobial peptides (AMPs) [29,30], and more efforts are needed to be done in this area.

## Figures and Tables

**Table 1.** Identification of fungal strains isolated from the gorgonian *Echinogorgia rebekka* sample based on morphological characteristics as well as DNA analysis of the internal transcribed spacer (ITS) region. Closest relatives to fungal strains according to BLAST search are presented.

**Strain**	**Morphological Identification**	**Seq. Length (bp)**	**Related Strain (BLAST)**	**Access. No.**	**Similarity (%)**	**Overlap (bp)**
WZL001	*Penicillium* sp.	559	*P. chrysogenum*	GU985086	98	554
WZL002	*Nigrospora* sp.	528	*Nigrospora* sp.	HQ248210	99	513
WZL003	*Alternaria* sp.	537	*Alternaria alternata*	GU325663	100	517
WZL004	*Aspergillus* sp.	545	*Aspergillus versicolor*	EU709775	99	537
WZL005	*Aspergillus* sp.	533	*Aspergillus* sp.	HQ637366	98	527
WZL006	*Nectria* sp.	543	*N. haematococca*	GU134901	98	521
WZL007	*Cladosporium* sp.	527	*Cladosporium* sp.	JF819134	100	509
WZL008	*Hypocrea* sp.	594	*H. lixii*	AY857235	99	577
WZL009	*Cladosporium* sp.	529	*C. sphaerospermum*	JF793522	99	518
WZL010	*Aspergillus* sp.	553	*A. flavipes*	EF669591	98	539
WZL011	*Cladosporium* sp.	526	*C. uredinicola*	FJ025160	99	509
WZL012	*Penicillium* sp.	558	*P. polonicum*	AF033475	98	545
WZL013	*Aspergillus* sp.	561	*A. westerdijkiae*	EF661428	98	548
WZL014	*Aspergillus* sp.	565	*Aspergillus* sp.	HQ731625	98	551
WZL015	*Cladosporium* sp.	529	*C. cladosporioides*	GU932679	99	518
WZL016	*Cladosporium* sp.	518	*C. cucumerinum*	GU594745	99	509
WZL017	*Penicillium* sp.	563	*P. crustosum*	HM037943	99	559
WZL018	*Penicillium* sp.	556	*P. glabrum*	GU372904	99	544

**Table 2.** Antibacterial activities of ethyl acetate (EtOAc) extracts from fermentation broth and mycelia of the gorgonian-derived fungal strains.

**Fungal Strain**				***B. subtilis***		***S. aureus***		***E. coli***		***M. tetragenus***		***V. anguillarum***	

**Class**	**Order**	**Genus**	**Strain**	**Broth**	**Mycelia**	**Broth**	**Mycelia**	**Broth**	**Mycelia**	**Broth**	**Mycelia**	**Broth**	**Mycelia**
Eurotiomycetes	Eurotiales	*Penicillium*	WZL001	−	−	−	−	−	−	++	−	−	−
			WZL012	−	−	−	++	−	−	−	−	−	−
			WZL017	−	+++	++	−	−	−	++	−	−	−
			WZL018	−	−	−	−	−	−	−	++++	−	−
		*Aspergillus*	WZL004	−	−	++	++	−	−	−	−	−	−
			WZL005	−	−	−	++	−	−	−	−	−	−
			WZL010	−	−	++	++	−	−	−	−	−	−
			WZL013	−	−	−	−	−	−	++	−	−	−
			WZL014	−	−	++	−	−	−	−	−	−	−
Dothideomycetes	Pleosporales	*Alternaria*	WZL003	−	−	−	+	−	−	−	−	−	−
	Capnodiales	*Cladosporium*	WZL007	−	−	++	−	−	−	+	−	−	−
			WZL009	−	++	+++	++	−	−	++	−	−	−
			WZL011	−	−	++	++	−	−	−	−	−	−
			WZL015	−	+++	++	−	−	−	++	−	−	−
			WZL016	−	−	++	−	−	−	−	−	−	−
Sordariomycetes	Trichosphaeriales	*Nigrospora*	WZL002	−	−	−	−	−	−	−	++	−	−
	Hypocreales	*Nectria*	WZL006	−	−	−	++	−	−	−	−	−	−
		*Hypocrea*	WZL008	−	−	−	−	++	−	++	−	++	−

Note: Growth inhibition diameters were used to define the categories of bacterial growth inhibition:

−, no inhibition was detected;

+, growth inhibition diameter less than 7 mm;

++, between 7 and 10 mm;

+++, between 10 and 15 mm;

++++, more than 15 mm. Assays were carried out in triplicates.
